# Percutaneous C-Arm Free O-Arm Navigated Biopsy for Spinal Pathologies: A Technical Note

**DOI:** 10.3390/diagnostics11040636

**Published:** 2021-04-01

**Authors:** Masato Tanaka, Sumeet Sonawane, Koji Uotani, Yoshihiro Fujiwara, Kittipong Sessumpun, Taro Yamauchi, Shinsuke Sugihara

**Affiliations:** 1 Department of Orthopaedic Surgery, Okayama Rosai Hospital, 1-10-25 Chikkomidorimachi, Minami Ward Okayama 702-8055, Okayama, Japan; drsumeet166@gmail.com (S.S.); coji.uo@gmail.com (K.U.); fujiwarayoshihiro2004@yahoo.co.jp (Y.F.); Kittipong@gmail.com (K.S.); ygitaro0307@yahoo.co.jp (T.Y.); 2 Department of Orthopaedic Surgery, Shikoku Cancer Center, 160 Ko Minamiumemotomachi, Matsuyama 791-0280, Ehime, Japan; ssugihar@shikoku-cc.go.jp

**Keywords:** C-arm free, O-arm navigation, percutaneous biopsy, spine tumor

## Abstract

Background: Percutaneous biopsy under computed tomography (CT) guidance is a standard technique to obtain a definitive diagnosis when spinal tumors, metastases or infections are suspected. However, specimens obtained using a needle are sometimes inadequate for correct diagnosis. This report describes a unique biopsy technique which is C-arm free O-arm navigated using microforceps. This has not been previously described as a biopsy procedure. Case description: A 74-year-old man with T1 vertebra pathology was referred to our hospital with muscle weakness of the right hand, clumsiness and cervicothoracic pain. CT-guided biopsy was performed, but histopathological diagnosis could not be obtained due to insufficient tissue. The patient then underwent biopsy under O-arm navigation, so we could obtain sufficient tissue and small cell carcinoma was diagnosed on histopathological examination. A patient later received chemotherapy and radiation. Conclusions: C-arm free O-arm navigated biopsy is an effective technique for obtaining sufficient material from spine pathologies. Tissue from an exact pathological site can be obtained with 3-D images. This new O-arm navigation biopsy may provide an alternative to repeat CT-guided or open biopsy.

## 1. Introduction

The incidence of skeletal metastasis is approximately 20%, with the spinal column as the most common location [1]. More than 10% of cancer patients develop symptomatic spinal metastasis, particularly involving the thoracic spine [2,3]. The most frequent metastasis to the spine is from lung cancer, followed by breast and prostate cancer [4]. If the primary site of cancer is known, the biopsy of metastatic spinal tumors is unnecessary [5]. However, biopsy is needed for accurate diagnosis when the primary site is unknown [6]. The proper treatment of spinal lesions depends on precise histological diagnosis.

The core needle biopsy (CNB) of spinal lesions under computed tomography (CT) guidance is a valuable and effective diagnostic tool to plan correct therapeutic strategy [7]. However, multiple CT scans may be required for this procedure for the confirmation of needle placement. Still sufficient material cannot be obtained sometimes as the bore of the needle is small, therefore, the diagnostic accuracy of this procedure remains around 80% [8]. Open biopsy may be needed in such situations which requires a relatively large skin incision and intraoperative confirmation of the biopsy site can be difficult if the target is very small. The difficulty of open biopsy further increase if the lesion is close to important organs such as the spinal cord, vital organs and great vessels. To perform spinal biopsy safely and precisely, we herein introduced a novel technique of C-arm free percutaneous spinal biopsy with an O-arm navigation.

## 2. Methods

Patient history. A 74-year-old man with a pathology of the T1 vertebra was referred to our hospital. The patient reported two months’ history of muscle weakness of the right hand and cervicothoracic pain.

Physical examination. On examination, the patient described pain, numbness and clumsiness of the right hand. He could walk without support and showed no spasticity of the legs. He reported cervicothoracic pain on movement and manual muscle test (MMT) scores were 3–4 for right hands.

**Figure 1:**
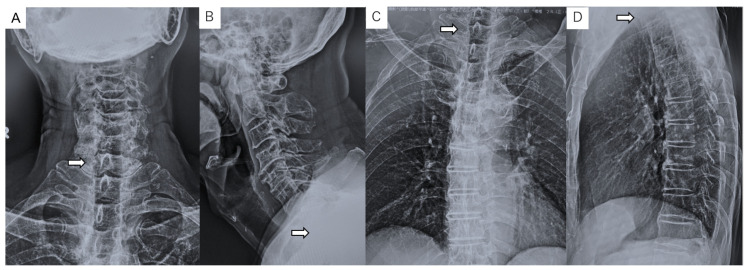
Radiograms of the patient show no pedicle winking owl sign at the right T1 vertebra: (**A**) cervical anteroposterior view; (**B**) cervical lateral view; (**C**) thoracic anteroposterior view; and (**D**) thoracic lateral view. White arrows indicate tumor location.

**Figure 2:**
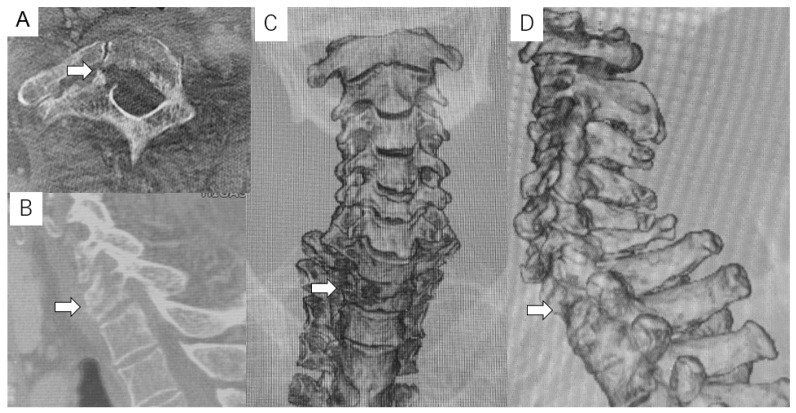
Computed tomography (CT) (**A**) Axial view—an osteolytic lesion was identified in the right T1 pedicle; (**B**) sagittal reconstruction view—the T1 vertebra collapsed; (**C**) anterior 3D—the right side of the T1 vertebra collapsed; and (**D**) lateral 3D. White arrows indicate tumor location.

**Figure 3:**
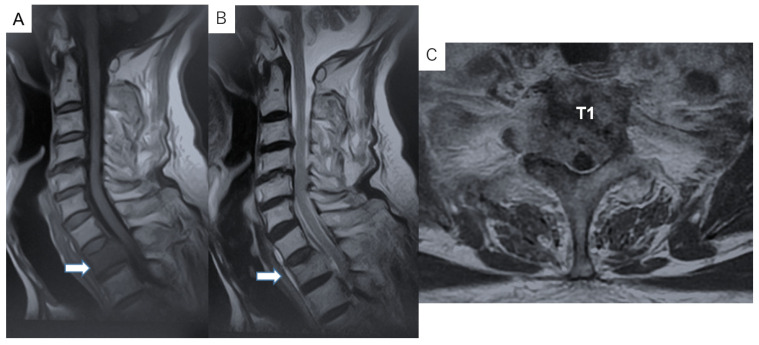
Magnetic resonance imaging (MRI) shows spinal cord compression by an epidural mass at the T1 level: (**A**) T1-weighted midsagittal image; (**B**) T2-weighted midsagittal image; and (**C**) T2-weighted axial image at the T1 vertebral level.

Preoperative imaging. Radiographs did not show a clear pedicle winking owl sign at the T1 vertebra (Figure 1). CT indicated an osteolytic lesion in the right T1 pedicle and T1 vertebral collapse. The reconstruction three-dimensional (3D) CT showed a collapse of the right side of the vertebral body and lateral mass in T1 vertebra (Figure 2). Magnetic resonance imaging (MRI) indicated spinal cord compression by an epidural mass at the T1 level (Figure 3).

**Figure 4:**
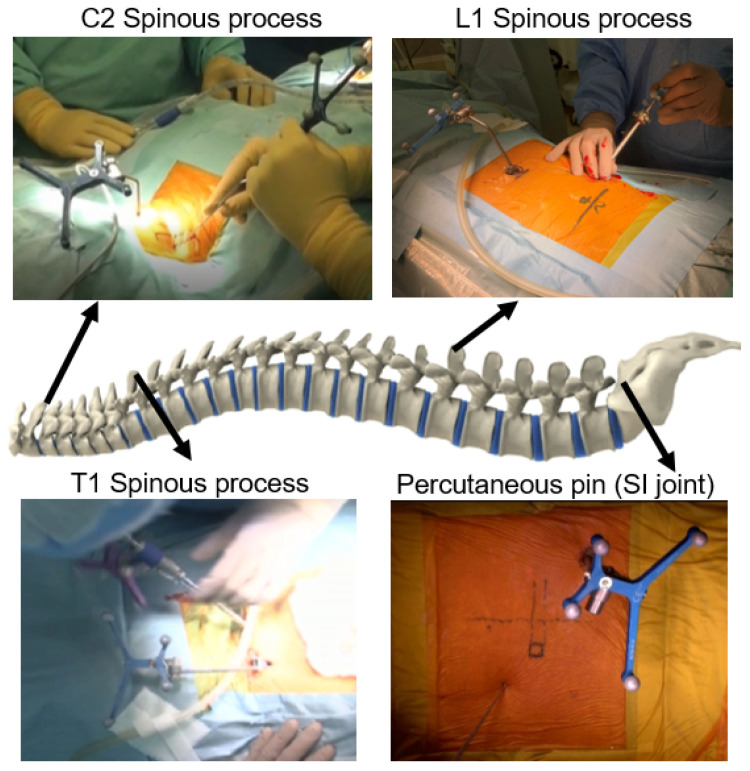
Position of the reference frame.

Navigated biopsy procedures. The patient is placed in a prone position under general anesthesia on a Jackson frame to perform the O-arm scan. A reference frame (RF) is attached to the spinous process near the targeted area. For the upper cervical spine and cervicothoracic junction, the C2 and C7/T1 spinous processes are the best RF sites, respectively, because these are prominent and allow the easy attachment of the RF. For the thoracolumbar junction and lumbar spine, the T12/L1 spinous processes and sacroiliac joint are good options for RF placement (Figure 4). 

**Figure 5:**
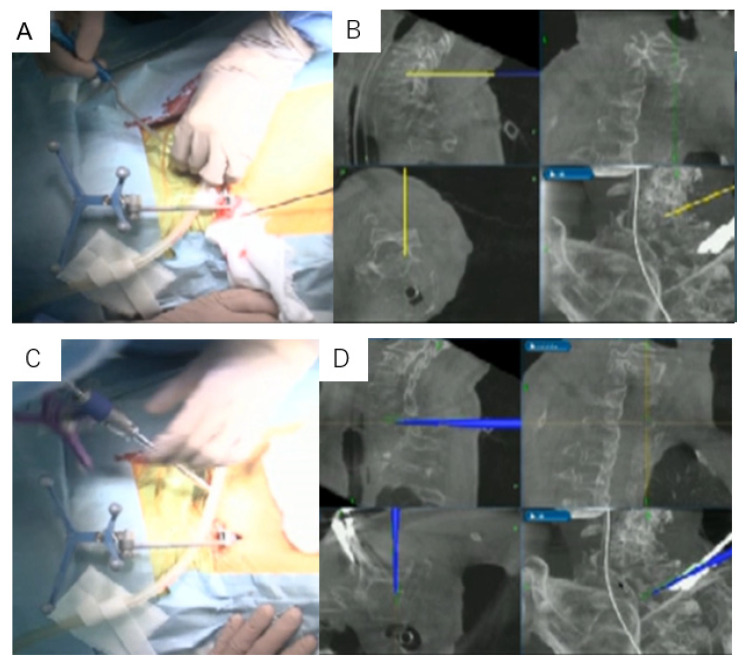
Skin incision and pedicle perforation under navigation: (**A**) the navigated pointer; (**B**) intraoperative navigation monitor; (**C**) the navigated pedicle probe; and (**D**) the intraoperative navigation monitor.

**Figure 6:**
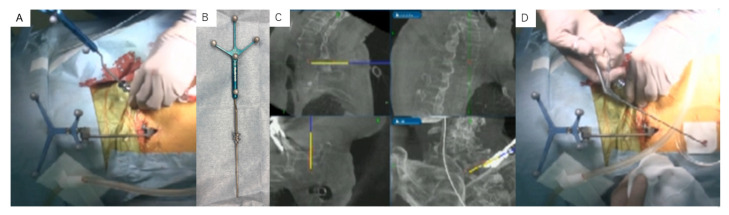
C-arm free O-arm navigated biopsy: (**A**) the tube insertion under navigation; (**B**) the small tube with a navigated probe; (**C**) intraoperative navigation monitor; and (**D**) the material from the biopsy.

The O-arm is then positioned and the reconstructed 3D images are obtained and transmitted to the Stealth Station navigation system Spine 7^®^ (Medtronic, Medtronic Sofamor Danek, Minneapolis, MN, USA). After every navigated spinal instrument is verified, the best entry point for the biopsy is marked by the navigated pointer (Figure 5A,B). A 5 mm skin incision is then made and the cortex of the targeted pedicle is penetrated by the navigated high-speed burr or navigated awl. A navigated pedicle probe is used to make a hole and reach the lesion (pedicle or vertebra) (Figure 5C,D). A small tube with a diameter of 5 mm and navigated tip is inserted (Figure 6A,B) and tumor tissue is obtained using micropituitary forceps (Figure 6C,D). In this procedure, the special non-navigated forceps is used whose tip passes only 8 mm beyond the tube. Thus, this maintains safety.

## 3. Results

CT-guided biopsy was performed for this patient, but diagnosis could not be obtained due to insufficient material. The patient then underwent C-arm-free O-arm navigated percutaneous spinal biopsy. After the O-arm navigated biopsy, small cell carcinoma was diagnosed. The patient was treated with chemotherapy and radiation without spinal decompression and fixation.

### Indications for Navigated Biopsy

The indications for this technique are spinal pathologies that include all the following conditions: lesion in the vertebra without known primary; failure of spinal CNB; and no other suitable biopsy site than the spine (Table 1).

## 4. Discussion

With the advent of MRI, more spinal pathologies can be diagnosed nowadays [9]. For the proper diagnosis of these pathologies, biopsy is needed. Various options available for biopsy are C-arm-guided, CT-guided and open biopsy. C-arm-guided biopsy is a good option but the diagnostic yield is around 56% for tumorous lesions [10]. Another problem with C-arm is that visibility in the high thoracic lesion is poor due to shoulder, scapula and lung shadows. In osteoporotic and obese patients, the C-arm image is poor and it is difficult to delineate bone shadows. Percutaneous CT-guided CNB is a modern method to obtain tissue from spinal pathologies. CNB under CT guidance is a fast, relatively simple, minimally invasive, and low-cost method for spinal lesions. However, CT-guided biopsy has disadvantages like a need for multiple scans for the proper placement of the needle, since there is no real-time image and it is not done in the sterile environment of operation room. The literature suggests that there is a risk of complications like neurological and vital organ injury in up to 26% cases in CT-guided biopsy [11,12,13]. The accuracy of this procedure has been reported as up to 80% [14,15]. If CT-guided CNB fails, another CT biopsy or open biopsy may be performed to obtain a sufficient amount of pathological material. It has been reported that the diagnosis was obtained in only 14% to 38% cases after repeat CNB for an initially nondiagnostic result [16,17]. The open biopsies of spinal tumors carry a potential risk of complications, such as massive bleeding, infection, damage to surrounding structures and a spread of pathology [18,19].

With our new technique, biopsy is performed by micro-forceps under local or general anesthesia and O-arm navigation guidance. A sufficient amount of material can thus be obtained. The risk of neural complications and organ injury can be reduced, because of the navigation. Performing this procedure does not require multiple CT scans. Thus, radiation exposure to the patient is reduced. We usually use a small field of view, low-dose mode for the O-arm 3D scan to reduce radiation to the patient. With this technique, we can reduce radiation exposure to operating room staff too [20]. 

This technique has some disadvantages. General anesthesia is preferable for this technique. Another disadvantage is that one more incision is needed for reference frame placement. If the reference frame is moved, the risk of navigation error is high [20]. Surgeons should therefore check the navigational accuracy; if there is any doubt regarding navigational accuracy, another O-arm scan should be taken.

## 5. Conclusions

O-arm navigation-guided biopsy provides sufficient tissue from an exact location for the accurate diagnosis of spinal pathologies. This procedure for spine biopsy reduces the risk of adverse events of intraoperative radiation to operation staff and patients. This new O-arm navigation biopsy may provide an alternative to repeat CT-guided or open biopsy.

## Figures and Tables

**Table 1:** Indications for navigated biopsy.

Category	
Primary lesion	Unknown
Tumor location	Lamina, pedicle, vertebral body, paravertebral mass
Core needle biopsy	Failure or impossible
Extra-spinal lesion	None or no suitable lesion for biopsy

## Data Availability

No new data were created or analyzed in this study. Data sharing is not applicable to this article.
